# Interleukin 16- (IL-16-) Targeted Ultrasound Imaging Agent Improves Detection of Ovarian Tumors in Laying Hens, a Preclinical Model of Spontaneous Ovarian Cancer

**DOI:** 10.1155/2015/567459

**Published:** 2015-06-16

**Authors:** Animesh Barua, Aparna Yellapa, Janice M. Bahr, Malavika K. Adur, Chet W. Utterback, Pincas Bitterman, Sanjib Basu, Sameer Sharma, Jacques S. Abramowicz

**Affiliations:** ^1^Departments of Pharmacology, Obstetrics and Gynecology, and Pathology, Rush University Medical Center, Chicago, IL 60612, USA; ^2^Department of Pharmacology, Rush University Medical Center, Chicago, IL 60612, USA; ^3^Department of Animal Sciences, University of Illinois at Urbana-Champaign, Urbana, IL 61801, USA; ^4^Poultry Research Farm, University of Illinois at Urbana-Champaign, Urbana, IL 61801, USA; ^5^Departments of Pathology and Obstetrics and Gynecology, Rush University Medical Center, Chicago, IL 60612, USA; ^6^Department of Preventive Medicine (Biostatistics), Rush University Medical Center, Chicago, IL 60612, USA; ^7^Departments of Pharmacology, and Obstetrics and Gynecology, Rush University Medical Center, Chicago, IL 60612, USA; ^8^Department of Obstetrics and Gynecology, Rush University Medical Center, Chicago, IL 60612, USA; ^9^Department of Obstetrics and Gynecology, Wayne State University, Detroit, MI 48201, USA

## Abstract

Limited resolution of transvaginal ultrasound (TVUS) scanning is a significant barrier to early detection of ovarian cancer (OVCA). Contrast agents have been suggested to improve the resolution of TVUS scanning. Emerging evidence suggests that expression of interleukin 16 (IL-16) by the tumor epithelium and microvessels increases in association with OVCA development and offers a potential target for early OVCA detection. The goal of this study was to examine the feasibility of IL-16-targeted contrast agents in enhancing the intensity of ultrasound imaging from ovarian tumors in hens, a model of spontaneous OVCA. Contrast agents were developed by conjugating biotinylated anti-IL-16 antibodies with streptavidin coated microbubbles. Enhancement of ultrasound signal intensity was determined before and after injection of contrast agents. Following scanning, ovarian tissues were processed for the detection of IL-16 expressing cells and microvessels. Compared with precontrast, contrast imaging enhanced ultrasound signal intensity significantly in OVCA hens at early (*P* < 0.05) and late stages (*P* < 0.001). Higher intensities of ultrasound signals in OVCA hens were associated with increased frequencies of IL-16 expressing cells and microvessels. These results suggest that IL-16-targeted contrast agents improve the visualization of ovarian tumors. The laying hen may be a suitable model to test new imaging agents and develop targeted anti-OVCA therapeutics.

## 1. Introduction

The global yearly rate of death of women due to ovarian cancer (OVCA) is approximately 140,200 women and that of the USA is approximately 15,000 [1, 2] making OVCA one of the lethal gynecological malignancies. Because of the lack of an effective early detection test, OVCA in most cases is detected at late stages. Development of resistance to currently available chemotherapeutics and frequent recurrences when detected at late stages decrease 5-year survival rate of OVCA patients to <20%. In contrast, OVCA can be cured in >90% cases when it is detected at early stage. Therefore, early detection of OVCA is crucial and an effective early detection test is urgently needed. Serum levels of CA-125 alone or in combination with traditional transvaginal ultrasound (TVUS) imaging are the currently available test for the detection of OVCA [3]. However, neither the CA-125 nor the TVUS can detect OVCA at early stage specifically as serum CA-125 level is elevated in patients with several benign gynecological as well as nongynecological abnormalities. On the other hand, although TVUS is the currently available preferred method for noninvasive imaging of ovarian abnormalities, unfortunately, with its limited resolution, traditional TVUS cannot detect OVCA at early stage [4]. In addition, a combination of serum CA-125 levels together with traditional TVUS imaging also failed to detect early OVCA as no imaging target in the ovary corresponding to the elevated serum CA-125 levels is established [4]. Thus a fresh approach is needed.

Extensive studies have been performed on the establishment of serum biomarkers for the detection of OVCA at early stage and a plethora of serum based marker(s) have been suggested. However, due to their lack of specificity and sensitivity, none of these markers was successful in detecting OVCA at early stage indicating that serum marker(s) alone may not be able to detect OVCA at early stage. Thus, an imaging target related to the malignant transformation of the ovary needs to be established and the current detection limit of traditional TVUS needs to be enhanced to detect early OVCA-related changes in the ovary. Moreover, to facilitate early detection of OVCA specifically, this imaging target(s) should also be associated with a surrogate maker(s) to be detectable in the serum. Contrast agents have been developed to enhance the visualization of tumors by several imaging modalities including TVUS scanning [5–9]. Imaging agents targeting *α*v*β*3-integrins and vascular endothelial growth factor receptor 2 (VEGFR-2) have been developed for contrast enhanced ultrasound imaging [10, 11]. However, very few reports are available on the ability of these targeted contrast agents in detecting OVCA at early stage. Moreover, absence of a corresponding serum surrogate marker reduces the specificity and sensitivity of these imaging agents. Thus additional imaging target(s) associated with malignant transformation needs to be established and imaging agents need to be developed to detect these new imaging targets for early detection of OVCA with high specificity.

Inflammation has been suggested as a risk factor for malignant transformation [12]. Unresolved inflammation leads to hypoxic conditions accompanied by changes in inflammatory cytokines including interleukin 16 (IL-16) [12, 13]. Ovulation is an inflammatory process which exposes ovarian surface (at the site of ovulatory rupture) and fimbrial epithelium (the site of reception of the ovulated ovum) to inflammatory factors including IL-16 secreted by immune cells. Exposure of the ovary and tubal epithelium to inflammatory agents due to frequent ovulation leads to the development of oxidative stress and longstanding unresolved oxidative stress has been suggested to cause malignant transformation. On the other hand, expression of IL-16 by the tumor epithelium and its serum levels has been reported to increase in association with ovarian tumor development [14, 15]. Moreover, IL-16 has also been reported as a proangiogenic factor [16] and may also be expressed by the endothelium of tumor-associated microvessels. Thus IL-16 represents a potential marker of early OVCA and IL-16 expressing tissues in the ovary can be detected by ultrasound imaging provided an IL-16-targeted ultrasound imaging agent can be developed.

Identification and access to patients with early stage OVCA are the significant barriers to develop and test the efficacy of contrast enhancing imaging agents in detecting spontaneous OVCA at early stage. Most of the available contrast agents were developed using rodents and thus are difficult to translate in human OVCA [11, 17–19], because rodents do not develop OVCA spontaneously and induced ovarian carcinomas in rodents are histopathologically not similar to those of spontaneous OVCA in humans [20]. Recently, laying hens have been shown to develop OVCA spontaneously with high incidence rates. Spontaneous OVCA in hens are remarkably similar to human OVCA with regard to tumor histopathology and expression of several molecular markers [14, 20–26]. Furthermore, methods for the imaging of hen ovaries and ovarian tumors by TVUS scanning have been adapted [27–29]. Moreover, similar to humans, expression of IL-16 by ovarian tumors has been reported to be increased in association with tumor development and progression in hens [14, 15]. Thus the laying hen represents a highly innovative model to test the feasibility of IL-16-targeted imaging agents for the detection of spontaneous OVCA at an early stage by noninvasive TVUS imaging. Therefore, the goal of this study was to examine whether IL-16-targeted contrast agents enhance the intensity of traditional TVUS imaging and improve the early detection of spontaneous ovarian tumors in laying hens, a preclinical model of OVCA.

## 2. Materials and Methods

### 2.1. Animals

A flock of 3-4-year-old commercial strains of White Leghorn laying hens (*Gallus domesticus*) were maintained under standard poultry care and management and provided with feed and water* ad libitum*. Egg laying rates of the hens were recorded on a daily basis. Egg laying rates in a hen are used as a relative indicator of ovulation rates in hens. The normal rate of egg laying by a commercial laying hen is more than 250 eggs per year and less than 50% of the normal laying rate is considered a low egg laying rate [27]. 150 hens with normal, low, or irregular egg laying rates and those that stopped laying with no large preovulatory follicle, with or without solid mass in the ovary and abdominal distention (a sign of possible ovarian tumor-associated ascites), were selected for IL-16-targeted contrast enhanced imaging agents. The incidence of ovarian cancer in laying hens of this age group was reported to be approximately 10% to 20% and is associated with low laying rates or complete cessation of egg laying [20, 21, 27]. All procedures were performed according to the Institutional Animal Care and Use Committee approved protocol.

### 2.2. IL-16-Targeted Contrast Enhanced Ultrasound Imaging Agents

IL-16-targeted imaging agents were prepared by conjugating anti-chicken IL-16 antibodies with Targestar containing microbubbles (Targeson, Inc., San Diego, CA). Targestar SA is a targetable ultrasound contrast agent coated with streptavidin. Biotinylated antibodies can be easily conjugated to the microsphere surface, enabling target-specific retention for molecular imaging. The agent remains acoustically active up to 15 minutes. Agents are administered as an intravenous bolus injection. Microbubbles preparation, ligand conjugation, characterization of labeled microbubbles, and their binding specificity of tumor tissues were similar to those reported earlier [10].

### 2.3. Ultrasound Imaging

#### 2.3.1. Precontrast Traditional Transvaginal Ultrasound (TVUS) Imaging

All hens were scanned using an instrument equipped with a 1 to 7.5 MHz transvaginal transducer (MicroMaxx, SonoSite, Inc., Bothell, WA) as reported previously with little modification [27, 29]. Hens were immobilized and gently restrained by an assistant. Transmission gel was applied to the surface of the transducer; the transducer was covered by a cover and to ensure uninterrupted conductance of the sound waves, gel was reapplied to the covered probe. The transducer was inserted approximately at a 30° angle to the body, 3 to 5 cm into the vagina, and 2-dimensional (2D) gray scale and pulsed Doppler sonography were performed. Young egg laying hens (as the ovaries of these hens contain more developing follicles compared to old hens) were used as standard controls for mechanical adjustment to reveal and characterize the fully functional normal ovaries of hens. The area of a tumor to be imaged was determined according to 3 conditions as reported previously [27, 29]: (a) the whole tumor, if possible, should be seen on the image; (b) the sectional plane should contain the solid part (wall, septa, and papillae) of the tumor; and (c) the most vascularized area was selected. For normal ovaries, ovaries without any detectable tumor, and atrophic ovaries, the region surrounding the ovary was scanned and the transducer was swept through the entire area for complete scanning of the ovary. Gray scale morphologic evaluation of the ovarian mass was performed with attention to the number of preovulatory follicles, the presence of abnormal-looking follicles, septations, papillary projections or solid areas, and echogenicity. After morphologic evaluation, color Doppler mode was activated for identification of vascular color signals. Once a vessel was identified on color Doppler imaging, pulsed Doppler was activated to obtain a flow velocity waveform.

#### 2.3.2. Injection of IL-16-Targeted Contrast Agents and Contrast Enhanced Ultrasound Imaging

Contrast imaging was performed following precontrast scanning. A preliminary experiment was conducted with IL-16-targeted or isotype control microbubbles using 10 animals containing fully functional ovaries to adjust the mechanical setup and determine the optimum dosage of microbubbles. The dose of 10 *μ*L/kg body weight was found optimal for better resolution in the preliminary experiment. Microbubbles containing contrast agents were prepared before injection. Briefly, the vial containing the microbubble suspension was inverted and gently rotated to resuspend the microspheres completely. The suspension was transferred from the vial by an injection syringe with a 19-gauge needle to a angiocatheter (small-vein infusion set, female luer, 12-in. tubing, 25-gauge needle; Kawasumi Laboratories, Tampa, FL) containing 100 *μ*L of 0.9% sodium chloride previously inserted into the left wing vein (brachial vein) of the hen and followed by the reloading of 100 *μ*L of a 0.9% sodium chloride solution. The loading of the sodium chloride solution before and after injection of microbubbles helped maintain the vascular patency and airtight condition, in addition to flushing the bubbles from the hen's circulation.

The area imaged during precontrast scanning was imaged again after contrast microbubble injection. Following injection of contrast agents and before postcontrast imaging, time was allowed for microbubbles to bind with their targets and retention of bounded microbubbles in the tumor as well as wash-out of unbound microbubbles. The timing of contrast imaging was determined through an initial experiment using different time points including 2, 5, 7, and 10 min. Imaging after 7 min of contrast agent injection was found optimum with minimum background signals. Then a destructive pulse was delivered and images were taken again. The difference in the intensity of ultrasound imaging between the images at 7 min after injection and images after the delivery of destructive pulse confirms that the signal acquired after contrast agent injection was from microbubbles-bounded target tissue. For an individual hen, the same imaging plane and same size of ROI were used for measuring the precontrast and postcontrast intensity of ultrasound imaging. All images (screenshots) were archived digitally in a still format as well as real-time clips on single-sided recordable digital video disks (DVD+R format; Maxell Corporation of America, Fair Lawn, NJ) readable on a personal computer.

#### 2.3.3. Evaluation of the Effects of IL-16-Targeted Contrast Agents

The effect of contrast agents was evaluated visually during the examination and the enhancement of tumor detection by contrast imaging was assessed afterward from reviewing the archived video clips. After reviewing the complete clip the image containing the stroma of normal hens or containing the tumor was selected and used as region of interest (ROI) for measuring the precontrast and postcontrast intensity of ultrasound imaging. In normal hens, areas containing large developing follicles were avoided during the selection of images containing the ROIs. The intensity of the pixels in the selected area was measured using a computer-assisted software program (MicroSuite version 5, Olympus Corporation, Tokyo, Japan) and expressed as arbitrary values. The intensity of the ROI (sum of the arbitrary values from the pixels within the region of interest) was measured from the precontrast and contrast image and expressed as the mean ± SD in 40,000-pixel area. The net contrast enhancement (CE = Ct − Cpt) was determined and the CE ratio (CER) was calculated using the following equation: CER = [(Ct − Cpt)/Cpt] × 100%, where Cpt = values from ROI of precontrast image and Ct = values from ROI of contrast image. As mentioned above, Ct is the difference between the intensity of ultrasound imaging from images taken at 7 min after injection of contrast agents and after the delivery of a destructive pulse.

### 2.4. Ovarian Gross Morphologic Evaluation

All hens were euthanized after contrast imaging and examined for the presence of a solid mass in the ovary as well as in any other organs, ascitic fluid, preovulatory follicles, and atrophy of the ovary, as reported previously [21]. Gross observation was compared with the sonographic evaluations and photographed. A normally functional ovary had viable preovulatory follicles (more detailed information on hen ovarian physiology has been published elsewhere [21, 27]), whereas no large follicles or visible lesions were found in normal hens that stopped egg laying. Tumor staging was performed according to the gross metastatic status as reported previously [21]. Briefly, early OVCA was characterized by detectable formation of solid tumor limited to the ovary. Late stages of OVCA were characterized by tumor metastasis to distant organs with moderate to extensive ascites.

### 2.5. Histologic Evaluation and Immunohistochemical Detection of Ovarian IL-16 Expressing Cells and Microvessels

Representative portions of a solid ovarian mass or the whole ovary (in cases of atrophic or grossly normal-appearing ovaries) were divided into several blocks, processed for paraffin or frozen sections, and stained with hematoxylin-eosin. Microscopic tumor (if present) in any part of the ovary was detected by routine histologic examination with hematoxylin-eosin staining, and tumor types were determined by light microscopy, as reported previously [21].

After histopathologic examination, paraffin sections (5 *μ*m thick) of normal and malignant ovaries of all stages and types were processed for routine immunohistochemistry to assess the frequency of IL-16 expressing cells and microvessels using rabbit anti-chicken IL-16 polyclonal antibodies as reported earlier [14, 15]. The frequencies of IL-16 expressing cells and microvessels were determined from the stroma of the ovarian tumors or ovarian stroma of normal hens (excluding the follicular areas), as reported earlier [28, 30] using a light microscope attached to digital imaging stereological software (MicroSuite version 5; Olympus Corporation) with little modification. Briefly, immunostained slides were examined at low-power magnification (×10 objective and ×10 ocular) to identify the areas with maximum IL-16 expressing cells or microvessels. Vessels with thick, regular, and complete muscular walls as well as vessels with large lumina were excluded from the count, as reported previously [28]. In each section, the 5 highly immunostained areas for IL-16 expressing cells or microvessels were chosen and immunopositive cells or microvessels (with leaky, incomplete, and thin vessel wall) were counted. The number of immunopositive cells or microvessels in a 20,000 *μ*m^2^ area was counted at ×40 objective and ×10 ocular magnification. The averages of these sections were expressed as the number of immunopositive cells or microvessels in a 20,000 *μ*m^2^ area of a normal ovary or ovary with tumor. Tumor histology and immunohistochemical observations were compared to the sonographic predictions.

### 2.6. Statistical Analysis

Descriptive statistics for imaging parameters were determined, and statistical analysis was performed in SPSS version 15 (SPSS Inc., Chicago, IL). The differences in the net intensities of ultrasound imaging and the frequencies of IL-16 expressing cells and microvessels among normal hens or hens with early and late stage OVCA were analyzed by the two-sample *t* test. The association between the intensity of ultrasound imaging and the frequency of IL-16 expressing cells or microvessels was examined by Pearson coefficient of correlations. *P* < 0.05 was considered significant. All reported *P* values are 2 sided.

## 3. Results

### 3.1. Evaluation of Noninvasive Contrast Enhanced Ultrasound Imaging

**Figure 1:**
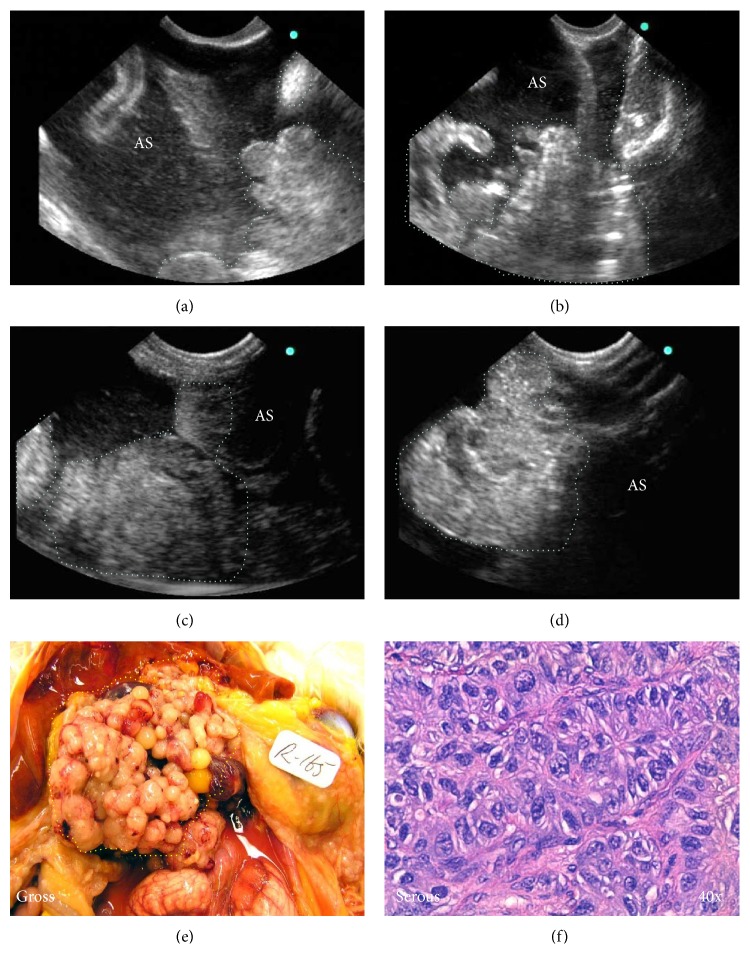
Enhancement of signal intensity of ultrasound imaging of hen ovarian tumors by IL-16-targeted contrast agents. (a) Case 1: precontrast gray scale ultrasonogram of a hen ovary showing solid mass (dotted lines) with septa and accompanied ascites (AS). (b) Postcontrast gray scale ovarian sonogram of the same hen showing enhanced visualization of the solid tumor mass. (c) Case 2: precontrast gray scale sonogram depicting a suspected ovarian mass (dotted lines) in another hen. (d) Gray scale sonogram of the same ovary (shown in (c)), depicting solid tumor mass with enhanced signal intensity after the injection of targeted imaging agents. (e) Gross presentation confirmed the imaging prediction of an ovarian tumor (shown in (c)-(d), appeared as cauliflower-shaped, yellow circled). (f) Histological examination showed a serous malignant tumor with cells containing large pleomorphic nuclei surrounded by a sheath of fibromuscular tissues. H&E staining.

**Figure 2:**
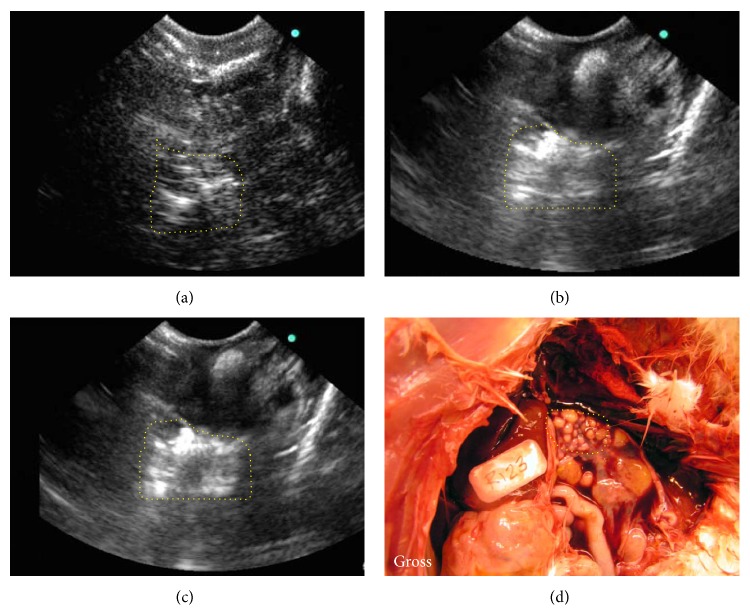
Detection of spontaneous ovarian tumors at early stage in hens by IL-16-targeted contrast enhanced ultrasound imaging. (a) Precontrast ovarian sonogram showing low intensity of ultrasound imaging. Presence of tumor-related solid mass in the ovary is inconclusive. (b)-(c) Corresponding contrast enhanced sonogram with enhanced visualization of ultrasound imaging at 5 min and 7 min after the injection of contrast agents, respectively, suggesting the presence of a small solid mass (yellow dotted lines) in the ovary. (d) Gross morphology shows the presence of a tissue mass (yellow dotted line) limited to a part of the ovary accompanied with a little ascites.

In normal hens with functional ovaries, multiple preovulatory follicles and small growing stromal follicles were observed on precontrast and contrast imaging. Compared to precontrast ovaries, visualization of solid ovarian masses with or without projected septa and papillary structures or both were enhanced remarkably in the ovaries of 23 hens. Of these 23 hens, 16 had solid masses in the ovary together with profuse ascites and were predicted to have late stage OVCA (Figures 1(a)–1(d)). In the remaining 7 hens, solid masses were limited to a part of the ovary with no or little ascites, and they were provisionally categorized as early stage OVCA (Figures 2(a) and 2(b)). Compared with precontrast scanning, IL-16-targeted contrast enhanced imaging improved the visualization of ovarian tumor masses in these 23 hens on gray scale (Figures 1 and 2). All of these hens were categorized as “hens with suspected ovarian cancer.”

All hens were euthanized following IL-16-targeted contrast imaging and sonographic predictions and stages of the tumor were confirmed by gross examination of hens at necropsy (Figures 1(e) and 2(d)). Ovarian morphology including ovarian follicles and their sizes, oviducts, presence of solid mass in the ovary, levels of tumor metastasis, OVCA stages, and accompanying ascites were recorded and tissues were processed as mentioned above. Tumor types were determined by routine hematoxylin and eosin staining (H&E) of paraffin sections (Figure 1(f)). Staging of ovarian tumors was performed as reported previously [21]. As observed during targeted imaging, late stage OVCA (*n* = 16 hens including 7 serous, 6 endometrioid, and 3 mucinous) was associated with moderate to profuse ascites and metastasized to peritoneal and abdominal organs. Tumors in early stage OVCA (*n* = 7 including 4 serous, 2 endometrioid, and 1 mucinous) were limited to the ovary with no or little ascites.

**Figure 3:**
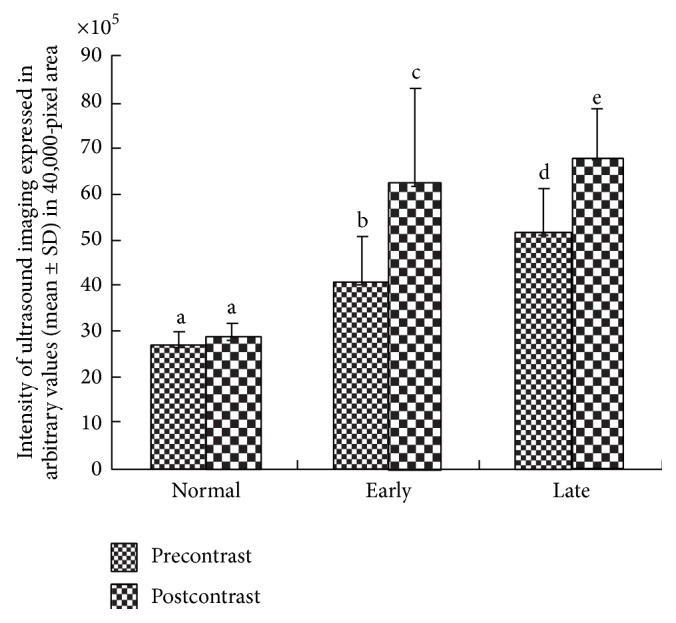
Changes in the signal intensity of ultrasound imaging by IL-16-targeted contrast agent in the ovary of laying hens with or without ovarian cancer (OVCA). Compared with precontrast imaging, IL-16-targeted contrast agents enhanced the intensities of ultrasound imaging significantly in hens with early stage OVCA as well as in late stage OVCA. However, significant differences were not observed between the pre- and postcontrast imaging in healthy hens. Different letters denote significant differences in the intensities of ultrasound imaging between the pre- and postcontrast imaging within the same group including hens with normal ovaries and with early and late stages of OVCA.

Overall, mean signal intensity (mean ± SD) of IL-16-targeted imaging in normal healthy hens with low egg laying rates was 27.7 × 10^5^  ±  3.3 × 10^5^ which was 1.06-fold higher than the precontrast signal intensities (Figure 3). However the difference was not statistically significant. On the other hand, compared with precontrast (39.9 × 10^5^  ±  10.8 × 10^5^) imaging, the mean signal intensity increased significantly (*P* < 0.05) to 61.9 × 10^5^  ± 21.2 × 10^5^ in postcontrast imaging in hens with tumor masses limited to the ovary (early stage). Thus, IL-16-targeted contrast enhanced imaging increased ultrasound signal intensity to 1.55-fold in hens with early stage OVCA (Figure 3). Similarly, in hens with late stage OVCA, the mean signal intensity (mean ± SD) increased significantly (*P* < 0.001) from 50.88 × 10^5^  ±  10.37 × 10^5^ in precontrast imaging to 67.89 × 10^5^ ± 10.86 × 10^5^ in postcontrast imaging (Figure 3). Pre- as well as postcontrast ultrasound signal intensities did not differ significantly among different histological subtypes of ovarian tumors.

### 3.2. Immunohistochemical Detection of IL-16 Expressing Cells and Microvessels

**Figure 4:**
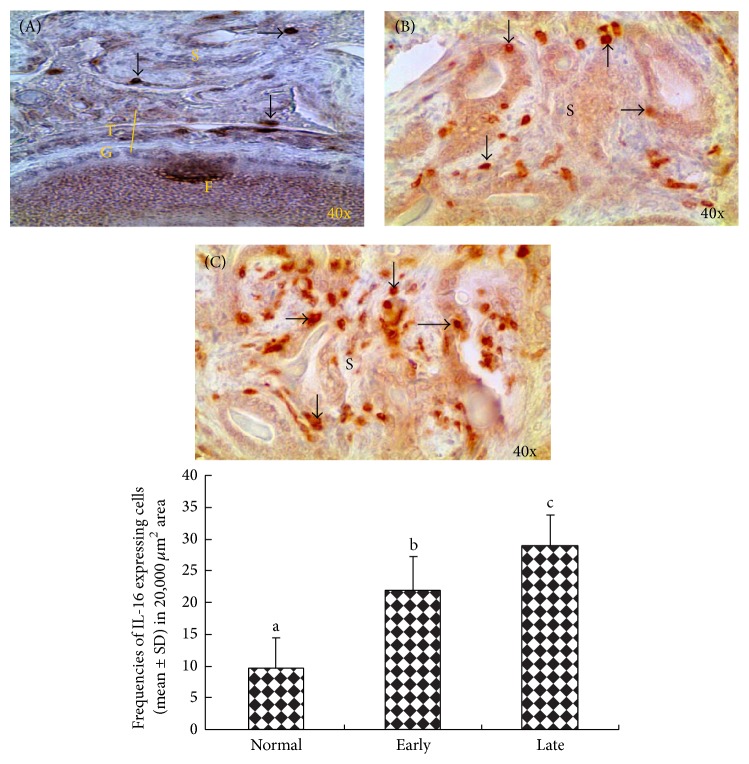
Immunohistochemical localization of IL-16 expressing cells in the ovaries of hens predicted to be normal or cancerous by IL-16-targeted contrast enhanced ultrasound imaging.* Top Panel*. (A) Section of a normal hen ovary showing few IL-16 expressing cells in the ovarian stroma (S) and the follicular (F) theca (T). (B)-(C) Sections of tumor ovaries at early (B) and late (C) stages of OVCA. Compared with normal ovary, many IL-16 expressing cells are seen in OVCA hens. S = stroma; arrows indicate the examples of IL-16 expressing cells.* Bottom Panel*. Compared with normal hens, the frequency of IL-16 expressing cells increased significantly (*P* < 0.001) with tumor development and progression to late stages. Bars with different letters indicate significant differences in the frequencies of IL-16 expressing cells among hens with normal, early stage, and late stage OVCA.

IL-16 expressing cells were detected in the stroma of normal or tumor-bearing ovaries and in the tumor vicinity including spaces between tumor glands (Figure 4, top panel). A number of epithelial cells (not all) in normal or tumor glands were also positive for IL-16 (Figure 4, top panel (B) and (C)). Very few IL-16 expressing cells were seen in the ovarian stroma and the follicular theca layer of normal healthy hens with low egg laying rates (Figure 4, top panel (A)). Compared with normal hens many IL-16 expressing cells were localized in hens with OVCA (Figure 4, top panel (B)-(C)). The frequency of stromal IL-16 expressing cells was significantly (*P* < 0.0001) higher in hens with early stage OVCA (mean ± SD = 21.85 ± 5.42 in 20,000 *μ*m^2^ of tumor tissue) than in normal hens (9.56 ± 4.87 in 20,000 *μ*m^2^ of ovarian stromal tissue) and increased further in hens with late stage of OVCA (28.56 ± 5.08 in 20,000 *μ*m^2^ of tumor tissue) (Figure 4, bottom panel).

**Figure 5:**
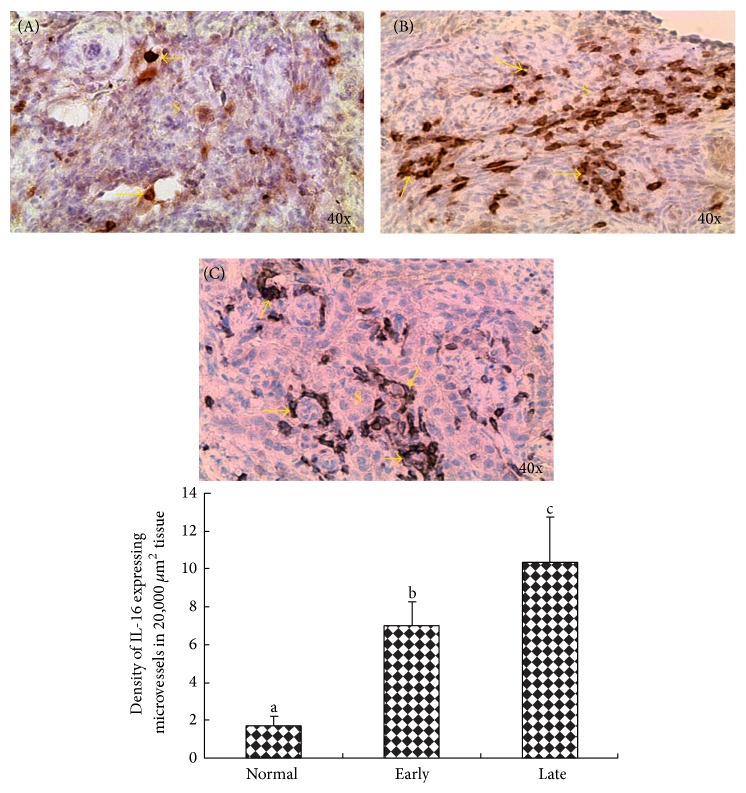
Expression of IL-16 by microvessels in the ovaries of hens with or without ovarian tumors scanned by targeted ultrasound imaging.* Top Panel*. (A) Section of a normal ovary showing few IL-16 expressing microvessels in the stroma (S). (B) and (C) Sections of malignant ovaries at early (B) and late (C) stages of OVCA. Compared with normal hens (A), more IL-16 expressing microvessels are seen in OVCA hens. S = stroma; arrows indicate examples of IL-16 expressing microvessels.* Bottom Panel*. Compared with normal hens, the frequency of IL-16 expressing microvessels was significantly (*P* < 0.001) high in OVCA hens at early and late stages. Bars with different letters indicate significant differences in the frequencies of IL-16 expressing microvessels among hens with normal, early stage, and late stage OVCA.

IL-16 expressing microvessels were detected in both normal ovaries and ovaries with tumor (Figure 5, top panel (A)–(C)). In normal ovaries, very few IL-16 expressing microvessels were seen in ovarian stroma (Figure 5, top panel (A)). Compared with normal ovary, many IL-16 expressing microvessels were localized in the stroma of ovaries with tumor (Figure 5, top panel (B)-(C)). The frequencies of IL-16 expressing microvessels were significantly (*P* < 0.0001) greater in hens with early stage OVCA (mean ± SD = 7.0 ± 1.29 in 20,000 *μ*m^2^ of tumor tissue) than in normal hens (1.71 ± 0.49 in 20,000 *μ*m^2^ of ovarian stromal tissue) and increased further (*P* < 0.0001) in hens with late stage of OVCA (10.33 ± 2.38 in 20,000 *μ*m^2^ of tumor tissue) (Figure 5, bottom panel). Differences in the frequencies of IL-6 expressing microvessels were not observed among different histological subtypes of malignant ovarian tumors in hens.

Increases in signal intensities due to IL-16-targeted contrast imaging were positively correlated with the frequencies of IL-16 expressing microvessels in ovarian tumors at early stage (*r* = 0.46) and late stage (*r* = 0.70). These results support the predictions of IL-16-targeted contrast imaging that enhanced signal intensity due to the contrast imaging in hens with tumors was due to the increased IL-16 expressing cells and microvessels in the ovaries with tumors.

## 4. Discussion

This study examined, for the first time, suitability of IL-16-targeted contrast agent, a newly developed ultrasound imaging agent, in improving the* in vivo* visualization of ovarian tumors in laying hens, a preclinical model of spontaneous OVCA. The results of this study demonstrated that IL-16-targeted contrast imaging agents bound with their targets expressed by ovarian tumors at early and late stages in hens and enhanced the intensities of ultrasound imaging signals from these tumors.

Increased expression of IL-16, a proinflammatory cytokine, has been reported to be associated with the development and progression of several malignancies including OVCA [14, 15, 31]. In addition to stromal cells of the tumor, tumor epithelium has also been reported to express IL-16 [14, 15]. Thus IL-16 expressing cells in ovarian tumors represent a potential target for ultrasound imaging for noninvasive detection of OVCA at early stage provided a targeted imaging agent is developed. In this study, compared with precontrast, IL-16-targeted contrast enhanced imaging increased the ultrasound signal intensity remarkably from hens with ovarian tumors at both early and late stages. These results suggest that IL-16-targeted contrast agents bound with their targets in the tumor tissues. In addition, as reported earlier for humans and hens [14, 15], this study also showed significant increase in the frequency of IL-16 expressing cells in hens with early and late OVCA compared to normal hens. Thus, higher signal intensities in hens with early and late stage OVCA than in normal hens may be, in part, due to the increased frequency of targets (IL-16 expressing cells) in OVCA hens which bound with their ligands (IL-16-targeted imaging agents).

IL-16 is a proangiogenic factor suggested to stimulate tumor-associated angiogenesis [16]. Furthermore, the frequency of IL-16 expressing cells was reported to be positively correlated with the frequencies of smooth muscle actin (SMA) expressing microvessels [14] during OVCA development and progression in hens. In this study, endothelial cells of microvessels expressed IL-16. Furthermore, this study also showed that the density of IL-16 expressing microvessels increased significantly with the development of OVCA and increased further as the tumor progressed to late stages. The frequencies of tumor-associated microvessels expressing *α*_v_ *β*_3_-integrins and VEGFR-2 have also been suggested to increase contrast enhanced ultrasound signal intensities [32, 33]. Thus, in addition to malignant cells, increase in the frequency of IL-16 expressing microvessel might also be a reason for the increased signal intensities during contrast enhanced imaging in hens with OVCA.

The results observed in the current study have, from translational point of view, some exceptional aspects. First, most of the contrast agents so far developed including two most extensively studied agents *α*_v_ *β*_3_-integrins and VEGFR-2 have limited success as expression of these targets was mainly limited to the blood vessels. Moreover, no corresponding serum markers of these imaging targets specific to OVCA have been established making their application difficult for early detection of OVCA. In contrast, in addition to the expression of IL-16 by the tumor epithelium and the microvessels, IL-16 is also secreted into the circulation. Serum levels of IL-16 have been reported to be increased significantly in association with OVCA development and progression [14, 15]. Thus, serum IL-16 levels offer a potential marker to be used in conjunction with IL-16-targeted contrast enhanced ultrasound imaging for the detection of OVCA at early stage. Second, most of the previous studies used rodent models with induced tumors. On the other hand, this study used laying hens, the only widely available and easily accessible spontaneous model of OVCA. Rodents do not develop OVCA spontaneously and the histopathology of induced OVCA is not similar to those of spontaneous OVCA. Moreover, anatomical differences in the location of induced rodent models (subcutaneous tumor) compared with deeper tissue like the ovary may also affect the transduction of ultrasound signals as well as the behavior of contrast agents. Thus information on the binding ability and detection of spontaneous OVCA by contrast agents (as seen for IL-16) is essential. Third, chickens are easy to access to test and develop targeted imaging agents as well as anti-OVCA drugs for the detection and treatment of spontaneous OVCA. Moreover, because of the lower cost of hens, this model is also suitable for toxicological studies of newly developed imaging agent or therapeutic in a cost-effective way. Presently, studies with hens are ongoing in which animals are being monitored prospectively with IL-16-targeted contrast agents together with serum IL-16 levels to detect spontaneous ovarian tumor development at relatively earlier stages. This study has also some limitations. We did not use animals with benign ovarian tumors. Small sample size specially the number of hens with ovarian tumors may also be a limitation of this study.

## 5. Conclusion

Overall, the results of the present study suggest that the IL-16-targeted contrast agents bind with their targets expressed by the spontaneous ovarian tumors in hens and enhance the visualization of tumors at early and late stages. This study also suggests that laying hens offer a new avenue for testing and development of new contrast agents and targeted antiangiogenic therapeutics.
